# Enhanced Osteogenic Response to an Osteochondral Scaffold Modified with BMP-2 or Strontium-Enriched Amorphous Calcium Phosphate in a Co-Culture In Vitro Model

**DOI:** 10.3390/jfb16080302

**Published:** 2025-08-21

**Authors:** Stefania Pagani, Manuela Salerno, Janis Locs, Jana Vecstaudza, Laura Dolcini, Milena Fini, Gianluca Giavaresi, Giuseppe Filardo, Marta Columbaro

**Affiliations:** 1 Surgical Sciences and Technologies, IRCCS Istituto Ortopedico Rizzoli, 40136 Bologna, Italy; gianluca.giavaresi@ior.it; 2 Applied and Translational Research Center, IRCCS Istituto Ortopedico Rizzoli, 40136 Bologna, Italy; manuelasalerno20@gmail.com; 3 Institute of Biomaterials and Bioengineering, Faculty of Natural Sciences and Technology, Riga Technical University, LV-1048 Riga, Latvia; janis.locs@rtu.lv (J.L.); jana.vecstaudza@rtu.lv (J.V.); 4 Baltic Biomaterials Centre of Excellence, Headquarters at Riga Technical University, LV-1048 Riga, Latvia; 5 Fin-Ceramica Faenza S.p.A, 48018 Faenza, Italy; ldolcini@finceramica.it; 6 Scientific Direction, IRCCS Istituto Ortopedico Rizzoli, 40136 Bologna, Italy; milena.fini@ior.it; 7 Faculty of Biomedical Sciences, Università della Svizzera Italiana, 6900 Lugano, Switzerland; giuseppe.filardo@eoc.ch; 8 Electron Microscopy Platform, IRCCS Istituto Ortopedico Rizzoli, 40136 Bologna, Italy; marta.columbaro@ior.it

**Keywords:** tissue regeneration, collagen, calcium phosphates, BMP-2, in vitro

## Abstract

**Background**: A trilayered collagen/collagen–magnesium–hydroxyapatite (Col/Col-Mg-HA) scaffold is used in clinical practice to treat osteochondral lesions, but the regeneration of the subchondral bone is still not satisfactory. **Objective**: The aim of this study was to test, in vitro, the osteoinductivity induced by the addition of bone morphogenetic protein-2 (BMP-2) or amorphous calcium phosphate granules with strontium ions (Sr-ACP), in order to improve the clinical regeneration of subchondral bone, still incomplete. **Methodology**: Normal human osteoblasts (NHOsts) were seeded on the scaffolds and grown for 14 days in the presence of human osteoclasts and conditioned medium of human endothelial cells. NHOst adhesion and morphology were observed with transmission electron microscopy, and metabolic activity was tested by Alamar blue assay. The expression of osteoblast- and osteoclast-typical markers was evaluated by RT-PCR on scaffolds modified by enrichment with BPM-2 or Sr-ACP, as well as on unmodified material used as a control. **Results**: NHOsts adhered well to all types of scaffolds, maintained their typical morphology, and secreted abundant extracellular matrix. On the modified materials, *COL1A1*, *SPARC*, *SPP1*, and *BGLAP* were more expressed than on the unmodified ones, showing the highest expression in the presence of BMP-2. On Sr-ACP-enriched scaffolds, NHOsts had a lower proliferation rate and a lower expression of *RUNX2*, *SP7*, and *ALPL* compared to the other materials. The modified scaffolds, particularly the one containing Sr-ACP, increased the expression of the osteoclasts’ typical markers and decreased the *OPG/RANKL* ratio. Both types of scaffold modification were able to increase the osteoinductivity with respect to the original scaffold used in clinical practice. BMP-2 modification seemed to be more slightly oriented to sustain NHOst activity, and Sr-ACP seemed to be more slightly oriented to sustain the osteoclast activity. These could provide a concerted action toward better regeneration of the entire osteochondral unit.

## 1. Introduction

The proper functioning of a joint requires a number of elements, such as the correct alignment of the joint ends, good lubrication, and, above all, the maintenance of the integrity of the tissues involved: cartilage and subchondral bone [1]. Several studies, mostly focused on the knee, have identified the role of cartilage and bone lesions in the onset of osteoarthritis (OA) and the correlation with disease progression [2,3,4]. Although this degenerative joint disorder has long been considered a disorder primarily of articular cartilage, the subchondral bone is now recognized as an important player [5,6,7,8]. It is therefore of paramount importance to adopt a therapeutic approach that addresses the entire osteochondral unit, including the subchondral bone [9].

One of the main strategies for the treatment of osteochondral lesions (OCL) involves the use of biomimetic scaffolds able to mimic the complexity of the osteochondral unit. Several materials have been developed for this purpose, but only a few have achieved implementation in clinical practice. Among these, a scaffold made of a collagen-based organic phase and a collagen comprising magnesium substituted-hydroxyapatite (Mg-HA) mineral phase (Col/Col-Mg-HA) has been in clinical use for years [10,11]. This material is stratified into three layers, each with distinct proportions of organic and inorganic phases, designed to mimic the extracellular matrix of articular cartilage, the tidemark zone, and subchondral bone tissue.

Mg-substituted HA provides more bone-like characteristics to the scaffold. Indeed, bone is the major site of storage of Mg ions, which are either adsorbed on the surface of apatite crystals or incorporated within the crystal structure. Mg-substituted HA should therefore enhance osteoblast differentiation and activity due to its affinity for the inorganic phase of natural bone [12].

The clinical use of Col/Col-Mg-HA showed a good ability to regenerate the cartilage layer and to significantly recover knee functionality up to a 10-year follow-up. Nevertheless, the regeneration of the bone component still remains insufficient, as emerged from the MRI analysis [13,14,15]. Enhancement of the layer designed for subchondral bone regeneration is critical to further improve performance of this composite material.

To achieve this aim, two distinct modifications have been designed for the subchondral layer in order to improve the regeneration of this joint’s component: the adsorption of bone morphogenetic protein 2 (BMP-2) and the addition of amorphous calcium phosphate (ACP) enriched with strontium (Sr) ions (Sr-ACP) [16,17].

BMP-2 is among the main regulators of bone formation, and its clinical use has been approved by the Food and Drug Administration (FDA). It promotes osteogenic differentiation by increasing alkaline phosphatase (ALP) activity, enhancing matrix mineralization, and recruiting stem cells or osteoblasts necessary for osteogenic initiation [16,18]. The absorption of this protein into the biomaterial allows local delivery at the lesion site, avoiding the possible side effects of systemic administration [19].

As a second strategy to enhance the osteoinductive potential of the subchondral bone-like scaffold layer, partially Sr-substituted ACP granules (Sr-ACP) were added to the Col/Col-Mg-HA matrix. This approach combines the pro-osteogenic characteristics of calcium phosphates and the anti-resorptive effects of Sr ions [20,21]. Although the benefits of incorporating Sr in different biomaterials have been confirmed in several studies, they have yet to be tested in combination with ACP in subchondral bone regeneration. Chemically, the incorporation of Sr into calcium-rich materials is possible due to the similar size and charge (+2) [17].

The in vitro osteogenic potential of these enhanced materials was recently assessed from multiple perspectives using two distinct models. One simulated the environment of aged and osteoarthritic joints by culturing primary human osteoblasts (NHOsts) on scaffolds under conditions rich in inflammatory mediators and reactive oxygen species. An aggressive microenvironment that mimics clinical conditions in which these scaffolds are most commonly implanted is key to reveal the cellular response in patients [22].

The second model focused on reproducing the complexity of the native tissue. Mesenchymal stromal cells (MSCs) were seeded on the materials in an indirect tri-culture model, including osteoclasts and human umbilical vein endothelial cells (HUVECs). A microenvironment enriched with paracrine factors secreted by various cell types offers a more physiologically relevant model for evaluating the osteogenic differentiation of MSCs [23,24,25,26].

Using a similar tri-culture model, we set out to study the behavior of more differentiated cells, osteoblasts, to obtain a more complete picture of the influence of enhanced Col/Col-Mg-HA materials on bone-forming and bone-resorbing cells. Taken together, these studies compose a useful framework for evaluating the novel biomaterials using different cell types and alternative microenvironments to cover several aspects.

The aim of this study was to build upon the ex vivo and in vivo findings of Xu et al. [16,17] by completing an investigation involving an in vitro co-culture approach with NHOsts, primary human osteoclasts, and conditioned medium from primary human endothelial cells to elucidate the effects of the modified scaffolds on these cell types.

## 2. Materials and Methods

### 2.1. Scaffolds

A material composed of 60% equine collagen/40% collagen–magnesium–hydroxyapatite (Col/Col-Mg-HA) (Maioregen, Fin-Ceramica, Faenza, Italy), with a size of Ø 8 mm and h 2 mm and mimicking the composition of the subchondral bone, was used as an osteochondral (OC) or control scaffold. The BMP-2-enhanced scaffold (OC+BMP-2) was obtained by absorbing BMP-2 (R&D System, Minneapolis, MN, USA) on the control material. Briefly, 4 µg BMP-2 dissolved in a few microliters was gently added dropwise on the top surface of each dry scaffold to provide an even distribution and avoid leaking. The scaffolds were then incubated for 30 min at 37 °C and immediately used in the experiments. The other experimental material (OC+Sr-ACP) was obtained by the addition of 100–150 µm sized Sr-ACP granules to the scaffold’s Col/Col-Mg-HA layer (30 w/w%). Synthesis of Sr-ACP, processing of the granules, and OC+Sr-ACP scaffold preparation were as previously described [17].

### 2.2. Cell Cultures

Normal human osteoblasts (NHOsts) were purchased from the American Type Culture Collection (ATCC, Rockville, Maryland, MD, USA) and expanded in an appropriate growth medium (GM) composed of Osteoblast Basal Medium (LONZA, Walkersville, MD, USA) supplemented with 10% fetal bovine serum (FBS) (LONZA). NHOsts were used at passage 4 for the experiments.

Human osteoclasts were obtained from Peripheral Blood Mononuclear Cells (PBMCs) derived from venous buffy coats of healthy adult male donors (Ethics Committee -CE AVEC- approval n. 191/2019/Sper/IOR, 04/19). PBMCs were isolated on a Ficoll-Hystopaque gradient (Sigma Aldrich, St. Louis, MO, USA) according to the manufacturer’s instructions and seeded at a density of 1 × 10^6^ cells/cm^2^ in Dulbecco’s modified Eagle medium (DMEM high glucose, Sigma Aldrich) supplemented with 10% FBS. After 24 h, non-adherent cells were discarded, and the medium was supplemented with 25 ng/mL of macrophage colony-stimulating factor (M-CSF), 30 ng/mL of receptor activator of NFkB factor ligand (RANKL), and 10^−7^ M of parathyroid hormone (PTH) (Peprotech, Rocky Hill, NJ, USA) (osteoclast differentiation medium). After 7 days, osteoclast differentiation was assessed by tartrate-resistant acid phosphatase (TRAP, Sigma Aldrich) staining, carried out according to the manufacturer’s instructions. Only differentiated osteoclasts were used for the co-culture experiments. All the cultures were maintained at 37 °C in a 5% CO_2_/95% air-controlled atmosphere.

Human umbilical vein endothelial cells (HUVECs, LONZA) were cultured in commercial endothelial growth medium (EBM-2 (endothelial cell basal medium-2)) supplemented with 2% FBS and the following growth factors: human Vascular Endothelial Growth Factor (hVEGF), human Epidermal Growth Factor (hEGF), human Fibroblast Growth Factor-Basic (hFGF-B), R3-Insulin-like Growth Factor (R3-IGF), Hydrocortisone, Heparin, Gentamicin, and ascorbic acid (EGM-2^TM^, LONZA). Conditioned medium from HUVECs at confluence was collected after a 24 h starvation period, centrifuged to remove cell debris, and immediately added to the NHOst/osteoclast co-culture.

### 2.3. Co-Culture Model

An indirect co-culture model of osteoblasts and osteoclasts was established using transwell polycarbonate membrane cell culture inserts on a 12-well plate (Thermo Fisher Scientific, Waltham, MA, USA). Osteoclasts were first seeded in the bottom well and allowed to differentiate for 7 days, as described in the previous paragraph. Then, the scaffolds (OC, OC+BMP-2, and OC+Sr-ACP) were placed in the transwell insert and preconditioned with 50 µL of GM for 1 h at 37 °C. NHOsts (2 × 10^5^ cells/scaffold, 30 µL of cell suspension) were then seeded on the scaffold surface and allowed to adhere for 2 h at 37 °C. Finally, 2 mL culture medium was added. The co-culture medium was 40% osteoblast differentiation medium (GM supplemented with 50 µg/mL of ascorbic acid, 7 mM of β-glycerophosphate, and 1 × 10^−7^ M dexamethasone, all from Sigma Aldrich) +40% osteoclast differentiation medium + 20% HUVEC conditioned medium. The medium was a mixture of culture media related to cell types and their respective seeding densities [24]. Medium was replaced twice a week. The cultures were evaluated after 7 and 14 days for metabolic activity and gene expression levels. Cell morphology and matrix synthesis were observed at the endpoint (14 days). The experiments were replicated three times using different cell batches.

### 2.4. Transmission Electron Microscopy (TEM) Analysis

At 14 days, NHOst cultured on the scaffolds were fixed with 2.5% glutaraldehyde in 0.1 M cacodylate buffer (Sigma Aldrich) for 1 h at room temperature, followed by for 3 h at 4 °C. Afterwards, the samples were washed with 0.1 M cacodylate buffer, post-fixed with osmium tetroxide (Electron Microscopy Sciences, Hatfield, PA, USA) for 2 h, dehydrated in graded concentrations of ethanol and propylene oxide (Sigma Aldrich), and finally embedded in EPON 812 (Electron Microscopy Sciences). Ultrathin cross sections (80 nm) were stained with uranyl acetate (Electron Microscopy Sciences) and lead citrate (Fluka Honeywell, NC, USA) and observed with a Jeol Jem-1011 electron microscope at 100 kV (Jeol LTD, Tokyo, Japan). Images were captured using an Olympus digital camera (Morada CCD camera, Olympus-Soft Imaging Solution GmbH, Münster, Germany) and iTEM software (Software: OSIS model iTEM, Olympus).

### 2.5. Alamar Blue Assay

Cell activity/viability of NHOsts grown on the scaffolds was evaluated by Alamar Blue dye (Thermo Fisher Scientific) after 7 and 14 days of culture. The constructs were first transferred to a new sterile culture multiplate to avoid the viability contribution given by osteoclasts; then, a mixture of dye and fresh culture medium in a 1:10 ratio was added to each well. After 4 h at 37 °C, fluorescence was read at 530ex–590em nm wavelengths using a Micro Plate reader (VICTOR X2030, Perkin Elmer, Milano, Italy) and expressed as relative fluorescence units (RFUs). Due to the composition and the porous structure of the materials, the dye tends to be absorbed. Therefore, a further 3 h incubation with only growth medium was performed to allow for the release of the retained reagent on the scaffold.

The values of fluorescence read after the second incubation were then added to the previously obtained results. The simple mixture of culture medium and dye was read as a blank value and subtracted to correct for the background fluorescence.

### 2.6. Gene Expression Analysis

Total RNA was separately isolated from NHOsts grown on the scaffolds and from osteoclasts grown on the bottom of wells after 7 and 14 days in co-culture. The expression of Runt-related transcription factor 2 (*RUNX2*), Transcription factor Sp7 (*SP7*), alkaline phosphatase (*ALPL*), osteonectin (*SPARC*), osteopontin (*SPP1*), osteocalcin (*BGLAP*), type 1 collagen (*COL1A1*), osteoprotegerin (*OPG*), receptor activator of nuclear factor kappa-Β ligand (*RANKL*), and Vascular Endothelial Growth Factor A (*VEGFA*) in NHOsts was evaluated. For osteoclasts, the expression of Associated Ig-Like Receptor (*OSCAR*), Cathepsin K (*CTSK*), and Acid Phosphatase 5 Tartrate Resistant (*ACP5*) was assessed. Briefly, 1 mL of cold TRIzol reagent (Ambion, Life Technologies, Carlsbad, CA, USA) was added to each sample and incubated for 5 min at room temperature. Chloroform was then added in a 1:5 ratio, and samples were centrifugated at 12,000 RCF at 4 °C for 15 min, after which the aqueous phase was collected, and an equal volume of cold 75% ethanol was added. Finally, purification step was performed using the Purelink™ RNA miniKit (Ambion, Life Technologies, Carlsbad, CA, USA) according to the manufacturer’s instructions. The RNA was then quantified by a spectrophotometer (NANODROP 2000, Thermo Scientific) and reverse transcribed using the Superscript Vilo cDNA synthesis kit (Life Technologies). Each sample was diluted to a final concentration of 5 ng/μL, taking into account the starting amount of RNA, to exploit the same range of amplification efficiency, and 10 ng of cDNA was tested in duplicate for each sample. Gene expression was evaluated by semiquantitative Real-Time PCR analysis (qPCR) using the SYBR green PCR kit (Qiagen GmbH, Hilden, Germany) in a Light Cycler 2.0 Instrument (Roche Diagnostics, GmbH, Manheim, Germany). The protocol included a denaturation cycle at 95 °C for 15 min, 25–40 cycles of amplification (95 °C for 15 s, an appropriate annealing temperature for each target for 20 s, and 72 °C for 20 s), and melting curve analysis to check for amplicon specificity. The mean threshold cycle was determined for each sample and used for the calculation of relative expression using the Livak method (2^−ΔΔCt^) [27], with glyceraldehyde-3-phosphate dehydrogenase (*GAPDH*) serving as the reference gene and the OC scaffold without modifications serving as the calibrator.

Detailed characteristics of primers are reported in Table 1.

### 2.7. Osteoclast Differentiation

To assess the differentiation of the mononucleated cells to the osteoclast lineage, as well as the response of osteoclasts to the materials, TRAP (tartrate-resistant acid phosphatase) staining was performed after 14 days of co-culture according to manufacturer’s instructions (Sigma-Aldrich, St. Louis, MO, USA). The positively stained cells showed different red intensity and multinucleation (Eclipse TiU, NIKON Europe BV, NITAL SpA, Milano, Italy).

### 2.8. Statistical Analysis

Statistical analyses were performed with GraphPad Prism software 9.5.1. Data are reported as mean ± standard deviations (SDs) at a significance level of *p* < 0.05. After having verified normal distribution and homogeneity of variance, a two-way ANOVA was performed, followed by Dunnett’s test to detect the significant differences among the modified biomaterials and controls at each timepoint, while Holm–Sidak’s multiple comparison test was performed to detect the significant differences among experimental times for the same scaffold. Cohen’s *d* values were reported to quantify the size effect of significant differences.

## 3. Results

### 3.1. Effects of the Modified Scaffolds on NHOst Morphology and Matrix Formation

**Figure 1:**
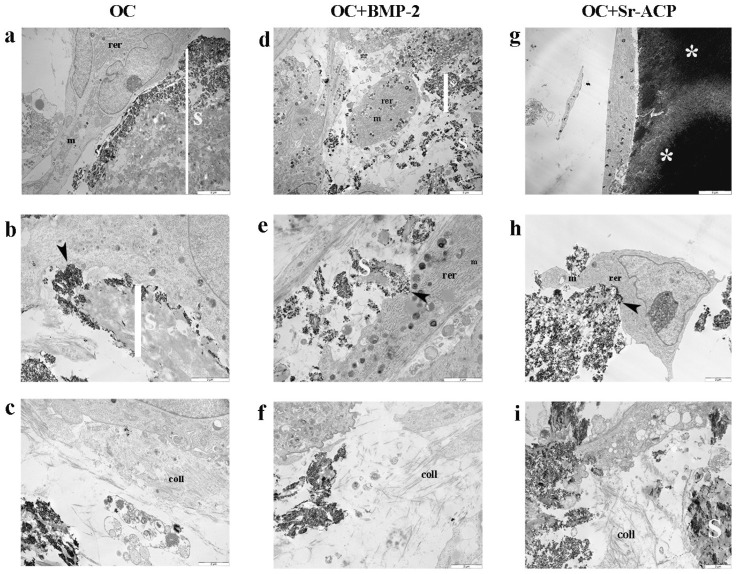
Morphology and matrix secretion of NHOsts grown on different OC scaffolds. Representative TEM images of NHOst/scaffold interaction and ECM deposition after 14 days of culture. rer: rough endoplasmic reticulum; m: mitochondria; coll: collagen fibrils; black arrowhead: focal contact; S: scaffold; white asterisk: ACP granule. (**a**,**d**,**g**) Scale bar: 5 μm; (**b**,**c**,**e**,**f**,**h**,**i**) scale bar: 2 μm.

Ultrastructural evaluation of the samples by TEM displayed a similar morphology between NHOst cells grown on modified and unmodified scaffolds (Figure 1). The cells exhibited the typical fusiform or cuboidal shape, with cytoplasm characterized by numerous well-preserved mitochondria (m) and abundant RER (rer), distinctive features of osteoblasts. In addition, we observed numerous focal contacts through which the cell membrane interacted with hydroxyapatite incorporated into the scaffolds or Sr-ACP granule surfaces, demonstrating that strong adhesion was promoted.

Again, from visual observations, we noted that similar amounts of collagen were secreted by the NHOst cells grown on the different materials. Collagen fibrils (coll) were randomly distributed and not oriented throughout the extracellular space in all the scaffolds, as is normally observed in the early stages of matrix deposition (Figure 1).

### 3.2. Alamar Blue Assay

**Figure 2:**
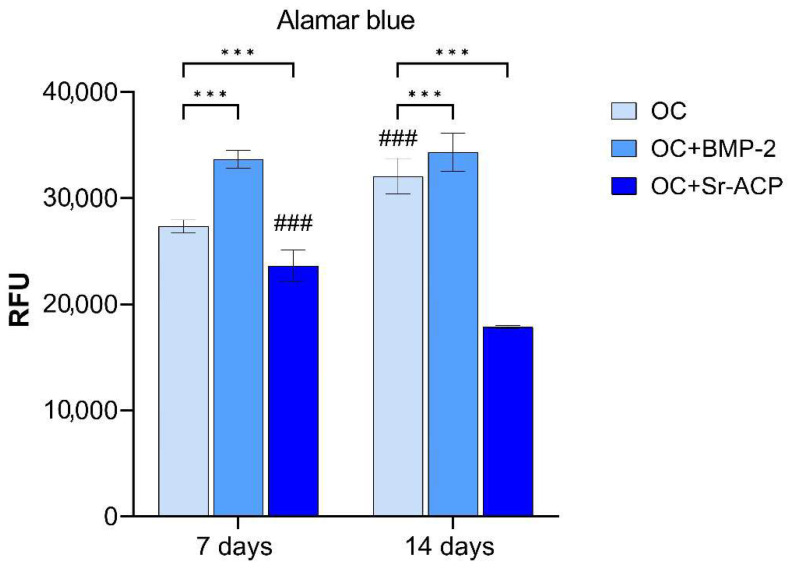
Cell proliferation of NHOsts grown on OC, OC+BMP-2, and OC+Sr-ACP scaffolds. The results were evaluated by Alamar Blue assay after 7 and 14 days of culture and expressed as relative fluorescent units (RFUs). Comparisons: *** *p* < 0.001 vs. OC; ^###^ *p* < 0.001 vs. day 7. Mean ± SD; *n* = 5 independent experiments with duplicates.

Regarding NHOst proliferation, evaluated by the Alamar Blue assay, significantly higher values were found for cells grown on OC+BMP-2 (7 d: *d* = 1221.5, *p* < 0.001; 14 d: *d* = 436, *p* < 0.001), and lower values were found for those grown on the OC+Sr-ACP (7 d: *d* = −712, *p* < 0.001; 14 d: *d* = −2734, *p* < 0.001) scaffold, compared to the unmodified material (OC), at both timepoints. At day 14, the proliferation was higher than at day 7 on the controls (*d* = 646, *p* < 0.001), but lower on the OC+Sr-ACP scaffold (*d* = −1110, *p* < 0.001). From day 7 to day 14, no further increase or decrease was observed for the OC+BMP-2 scaffold (Figure 2).

### 3.3. Effects of the Modified Scaffolds on Gene Expression

#### 3.3.1. Effects of the Modified Scaffolds on NHOsts

**Figure 3:**
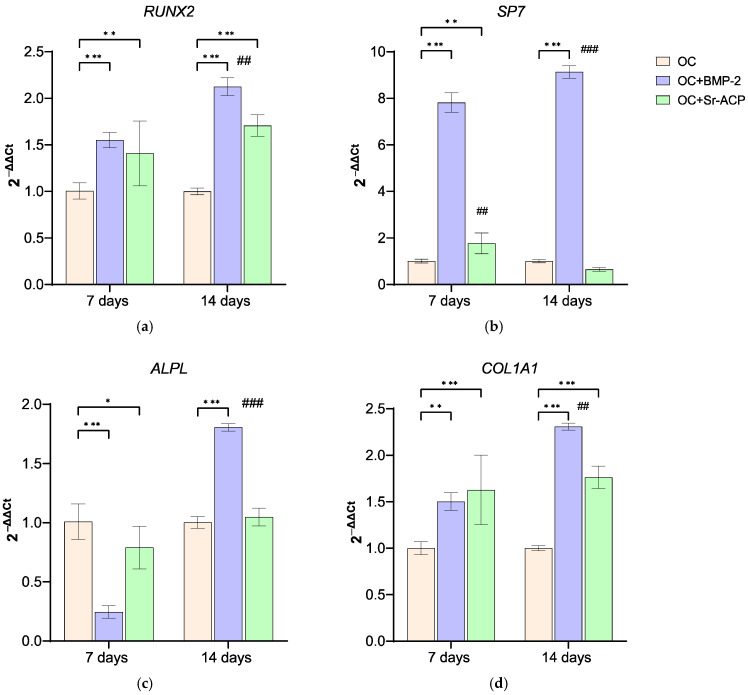
Expression of genes involved in activity of NHOsts cultured on scaffolds. *RUNX2* (**a**), *SP7* (**b**), *ALPL* (**c**), and *COL1A1* (**d**) expression levels after 7 and 14 days of culture on OC, OC+BMP-2, and OC+Sr-ACP scaffolds. The results have been normalized to *GAPDH* and are expressed as fold change relative to the control group (OC), considered as 1. Comparisons: * *p* < 0.05, ** *p* < 0.005, and *** *p* < 0.001 vs. OC at each timepoint. ^##^ *p* < 0.005; ^###^ *p* < 0.001 vs. 7 days. Mean ± SD; *n* = 3 duplicates.

The expression of *RUNX2* was significantly higher for NHOsts grown on OC+BMP-2 and OC+Sr-ACP scaffolds than on control scaffolds at both timepoints (7 d: OC+BMP-2, *d* = 2.7, *p* < 0.001; OC+Sr-ACP, *d* = 2.0, *p* < 0.005; 14 d: OC+BMP-2, *d* = 5.5, *p* < 0.001; OC+Sr-ACP, *d* = 3.5, *p* < 0.001). At 14 days, *RUNX2* was further increased compared to 7 days on OC+BMP-2 (*d* = 2.8, *p* < 0.005). *SP7* was much more markedly expressed by cells on OC+BMP-2 scaffolds than on OC scaffolds at 7 and 14 days of culture (*d* = 20.5, *d* = 24.5, *p* < 0.001), also increasing its expression over time (*d* = 4, *p* < 0.001), while in cells on OC+Sr-ACP, its expression was higher than on OC scaffolds only at 7 days (*d* = 2.3, *p* < 0.005), decreasing over time (*d* = −3.4, *p*< 0.005). *ALPL* expression, conversely, was lower on both experimental scaffolds compared to controls at day 7 (OC+BMP-2, *d* = −5.7, *p* < 0.001; OC+Sr-ACP, *d* = −1.6, *p* < 0.05), but it increased at day 14, reaching a value higher than on OC only on OC+BMP-2 (*d* = 6, *p* < 0.001), with a significant increase being recorded over time (*d* = 11.6, *p* < 0.001). *COL1A1* was upregulated on both experimental materials at both endpoints (7 d: OC+BMP-2, *d* = 2.40, *p* < 0.005, OC+Sr-ACP, *d* = 3.0, *p* < 0.001; 14 d: OC+BMP-2, *d* = 6.26, *p* < 0.001; OC+Sr-ACP, *d* = 3.65, *p* < 0.001), with an increase between days 7 and 14 on OC+BMP-2 (*d* = 3.8, *p* < 0.005) (Figure 3).

**Figure 4:**
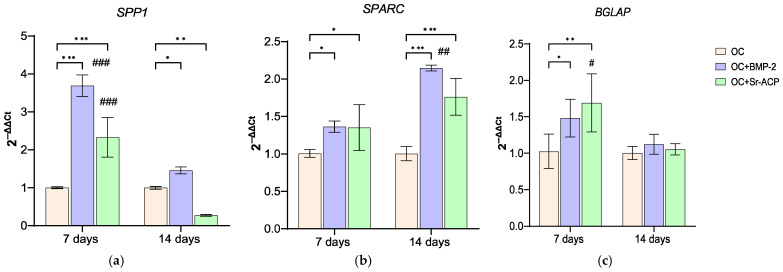
Expression of genes involved in the activity of NHOsts cultured on scaffolds. *SPP1* (**a**), *SPARC* (**b**), and *BGLAP* (**c**) expression levels after 7 and 14 days of culture on OC, OC+BMP-2, and OC+Sr-ACP scaffolds. The results have been normalized to *GAPDH* and are expressed as fold change relative to the reference group (OC), considered as 1. Comparisons: * *p* < 0.05, ** *p* < 0.005, and *** *p* < 0.001 vs. OC at each timepoint; ^#^ *p* <0.05, ^##^ *p* <0.005, ^###^ *p* < 0.001 vs. 7 days. Mean ± SD; *n* = 3 duplicates.

The expression of bone matrix-associated proteins was also evaluated. *SPP1* was significantly upregulated on OC+BMP-2 (*d* = 11, *p* < 0.001) and OC+Sr-ACP (*d* = 5.5, *p* < 0.001) compared to OC at day 7, but its expression decreased at day 14 (OC+BMP-2, *d* = −9.2, *p* < 0.001; OC+Sr-ACP, *d* = −8.5, *p* < 0.001), with OC+Sr-ACP reaching levels lower than OC (14 d: OC+BMP-2, *d* = 1.9, *p* < 0.05; OC+Sr-ACP, *d* = −3, *p* < 0.005). As observed for *RUNX2* and *COL1A1*, *SPARC* was expressed at higher levels by osteoblasts seeded on both enhanced materials at both timepoints (7 d: OC+BMP-2, *d* = 1.8, *p* < 0.05; OC+Sr-ACP, *d* = 1.7, *p* < 0.05; 14 d: OC+BMP-2, *d* = 5.6, *p* < 0.001; OC+Sr-ACP, *d* = 3.7, *p* < 0.001), with an increase over time on OC+BMP-2 being noted (*d* = 3.9, *p* < 0.005). *BGLAP* expression was significantly higher on the enhanced scaffolds at day 7 (OC+BMP-2, *d* = 1.9, *p* < 0.05; OC+Sr-ACP, *d* = 2.7, *p* < 0.005), but it decreased in a significant manner after 14 days on OC+Sr-ACP (*d* = −2.6, *p* < 0.05) (Figure 4).

#### 3.3.2. Effects of the Modified Scaffolds on NHOst Interaction with Osteoclasts and Endothelial Cells

**Figure 5:**
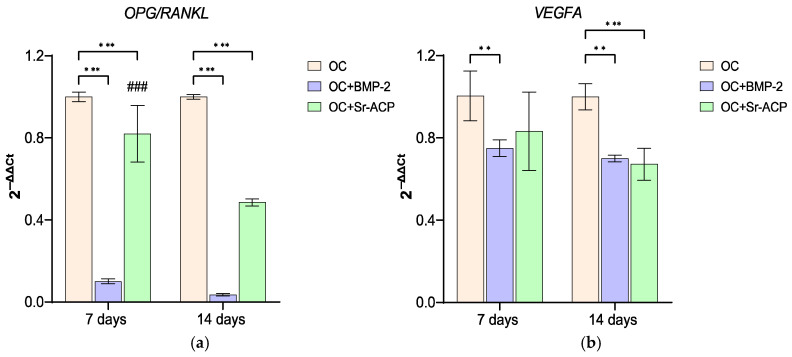
Expression of genes involved in osteoclast induction and angiogenesis in NHOsts cultured on scaffolds. *OPG/RANKL* (**a**) and *VEGFA* (**b**) expression levels after 7 and 14 days of NHOst culture on OC, OC+BMP-2, and OC+Sr-ACP scaffolds. The results have been normalized to *GAPDH* and are expressed as fold change relative to the reference group (OC), considered as 1. Comparisons: ** *p* < 0.005, and *** *p* < 0.001 vs. OC at each timepoint; ^###^ *p* < 0.001 vs. 7 days. Mean ± SD; *n* = 3 duplicates.

The *OPG/RANKL* ratio is often used as an indicator of the interaction between osteoblasts and osteoclasts. In this study, *OPG/RANKL* showed the highest values on the unmodified material (OC). On OC+BMP-2, the *OPG/RANKL* ratio was lowest at both timepoints (7 d: OC+BMP-2, *d* = −13.7, *p* < 0.001; OC+Sr-ACP, *d* = −2.7, *p* < 0.001; 14 d: OC+BMP-2, *d* = −14.7, *p* < 0.001; OC+Sr-ACP, *d* = −7.9, *p* < 0.001), while on OC+Sr-ACP, the ratio decreased over time (*d* = −5.1, *p* < 0.001). The expression of the angiogenic mediator *VEGFA* was partially inhibited in the osteoblasts seeded on the enhanced materials at both timepoints in comparison to OC (7 d: OC+BMP-2, *d* = −2.5, *p* < 0.005; 14 d: OC+BMP-2, *d* = −2.9, *p* < 0.005; OC+Sr-ACP, *d* = −3.2, *p* < 0.001) and remained stable over time (Figure 5).

#### 3.3.3. Effects of the Modified Scaffolds on Osteoclasts

**Figure 6:**
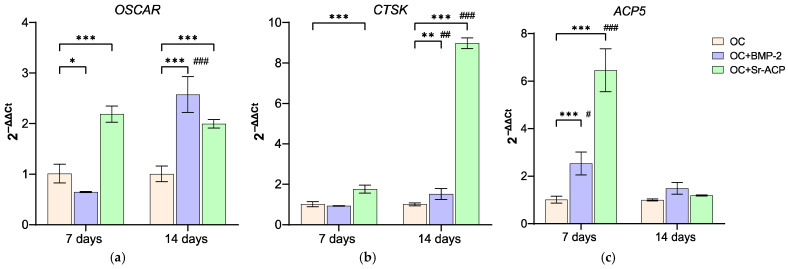
Expression of genes related to osteoclasts. *OSCAR* (**a**), *CTSK* (**b**), and *ACP5* (**c**) expression levels after 7 and 14 days of co-culture with NHOsts. The results have been normalized to *GAPDH* and are expressed as fold change relative to the reference group (OC), considered as 1. Comparisons: * *p* < 0.05, ** *p* < 0.005, and *** *p* < 0.001 vs. OC at each timepoint; ^#^ *p* <0.05, ^##^ *p* <0.005, ^###^ *p* < 0.001 vs. 7 days. Mean ± SD; *n* = 3 duplicates.

**Figure 7:**
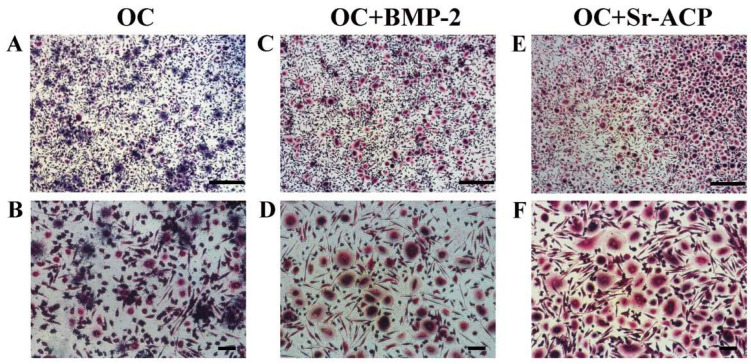
Representative images of differentiating osteoclast cultures stained for TRAP after 14 days of co-culture. Osteoclasts in presence of OC (**A**,**B**), OC+BMP-2 (**C**,**D**), and OC+Sr-ACP scaffold (**E**,**F**). Images at 4× (**A**,**C**,**E**) and 10× magnification (**B**,**D**,**F**). Scale bars: 500 μm (**A**,**C**,**E**) and 100 μm (**B**,**D**,**F**).

The influence of the enhanced materials on osteoclast differentiation and activity was assessed by the expression of *OSCAR*, *CTSK*, and *ACP5. OSCAR*, which contributes to osteoclast differentiation, as well as *RANKL*, was more expressed in OC+Sr-ACP at both timepoints compared to the control material (7 d: *d* = 6.8, *p* < 0.001; 14 d: *d* = 5.7, *p* < 0.001), while OC+BMP-2 showed its stimulating role on this gene after 14 days (7 d: *d* = −2.1, *p* < 0.05; 14 d: *d* = 9, *p* < 0.001), with a significant increase over time (*d* = 11.1, *p* < 0.001). Similarly, *CTSK* was upregulated in OC+Sr-ACP at both day 7 and day 14 (7 d: *d* = 3.9, *p* < 0.001; 14 d: d = 42, *p* < 0.005), while for OC+BMP-2, this only occurred at the 2-week timepoint (*d* = 2.71, *p* < 0.005). *CTSK* weakly increased over time on OC+BMP-2 (*d* = 3.1, *p* < 0.005), thus reaching a significant difference compared to cells on OC, but it experienced a very strong increase on OC+Sr-ACP (*d* = 38, *p* < 0.001). Finally, *ACP5* was more expressed on both enhanced materials than on OC after 7 days (7 d: OC+BMP-2, *d* = 3.8, *p* < 0.001; OC+Sr-ACP, *d* = 13.4, *p* < 0.001), while all scaffolds behaved similarly at the endpoint, decreasing at 14 days (OC+BMP-2: *d* = −2.6, *p* < 0.05; OC+Sr-ACP: *d* = −13, *p* < 0.001) (Figure 6). TRAP staining confirmed the gene expression results, showing a greater number of more differentiated cells in the presence of the enhanced materials, especially with OC+Sr-ACP (Figure 7).

## 4. Discussion

The positive clinical results of osteochondral scaffolds consisting of collagen and magnesium–hydroxyapatite are still limited by the incomplete regeneration of the subchondral bone layer. Therefore, we modified this part of the material (consisting of 60% equine collagen/40% collagen–magnesium–hydroxyapatite) by using two distinct approaches: the addition of BMP-2 or the addition of Sr-ACP. The aim was to ameliorate the biological responses of the cells in the subchondral layer and thus further improve the clinical performance of this biomaterial.

The strength and novelty of this work lies in the adopted model. Indeed, a culture system that includes osteoblasts and osteoclasts, in the presence of an endothelial cell supernatant, provides a more physiological microenvironment. Bone tissue homeostasis is governed by a complex mechanism primarily driven by the crosstalk between osteoblasts and osteoclasts. In this system, the intercellular communication is mediated through the secretion of specific proteins that activate bidirectional signaling pathways. Therefore, results from co-culture systems are more robust than those obtained from single cultures, and consequently, the observations made on biomaterials are more reliable.

The main finding emerging from this study is that both osteochondral scaffold augmentation strategies promote bone formation. With an in vitro model based on different cell types, we also demonstrated that BMP-2 mainly affected the activity of osteoblasts, whereas Sr-ACP mainly influenced osteoclasts.

BMP-2 is a known osteoinductive growth factor that has been largely investigated in vitro. Most studies, however, have been conducted on mesenchymal stromal cells rather than on osteoblasts. BMP-2 has been demonstrated by several authors to promote the expression of *COL1A1*, *ALPL*, and *BGLAP* genes and proteins in mesenchymal cells [28], but also to upregulate the expression of *SPP1* and *BGLAP* and to induce mineralization [29]. Here, we observe similar results with osteoblasts. The ability of the Col-Mg-HA layer to retain BMP-2 and release it gradually, as observed by Xu J. [16], could suggest that this growth factor acts primarily through direct contact between material and cells.

On OC+Sr-ACP scaffolds, the expression of osteogenic “key markers” such as *COL1A1*, *SPP1*, *SPARC*, and *BGLAP* was, however, higher than on OC at different timepoints. Conversely, *ALPL* never exceeded the OC values, supporting the hypothesis of a more differentiated state of osteoblasts on this scaffold.

In vitro and in vivo studies have long since demonstrated the effectiveness of calcium phosphate in bone regeneration [30,31,32]. Calcium phosphates have also been functionalized with the addition of a variety of ions, organic molecules, and drugs, which makes this compound a potential delivery system able to better conduct and modulate bone tissue restoration [33]. From this perspective, OC+Sr-ACP is also thought to be a suitable tool for improving the Col/Col-Mg-HA material. The Sr ion is recognized to stimulate osteogenesis, and its presence in our modified material could have contributed to the good response observed in osteoblasts, similarly to what has been reported by others [34,35]. We initially hypothesized that the promotion of bone regeneration would be paralleled by the inhibition of osteoclast activity, making its use suitable in conditions such as osteoporosis [36,37]. Conversely, in our co-culture study, we demonstrated a higher osteoclast activation state on this scaffold, mediated by the expression of *OSCAR* and *ACP5*, after only 7 days.

In summary, the direct comparison of these two bone scaffold modifications in this co-culture model revealed interesting insights into the differential mechanisms underlying increased bone formation, at the same time confirming what was observed in the aforementioned in vivo studies of Xu.

Osteoblasts are the bone cellular component responsible for matrix deposition and calcification through synthesis and secretion of structural proteins such as type 1 collagen, osteopontin, osteonectin, osteocalcin, and enzymes such as alkaline phosphatase, responsible for the mineralization process and the deposition of hydroxyapatite crystals during bone formation [38,39]. In general, all these markers were more expressed on materials containing BMP-2 or Sr-ACP compared to OC control. *COL1A1* and *SPARC* showed a similar trend, with a more noticeable increase over time in the presence of BMP-2.

When in contact with the different materials, osteoblasts showed a well-preserved ultrastructure, as indicated by good mitochondrial morphology, abundant RER, and numerous focal contacts. Optimal adhesion was appreciable on all three scaffolds, in particular in correspondence with the HA crystals. Also, no significant differences in the amount of banded collagen secreted were observed among the different materials, thus confirming osteoblasts’ good overall status. However, lower values of cell proliferation were observed on OC+Sr-ACP by Alamar Blue testing. A previous study of cytotoxicity, performed in accordance with ISO 10993-1, quantified the cell viability of osteoblast Balb/c 3T3 by using extracts from the simple scaffold, or from ACP or Sr-ACP scaffold, at increasing dilutions from 100% to 6.4%. In that model, partially different from ours, the cell viability results were lower compared to a negative control, consisting of cells in growth medium, at the highest extract concentration [17]. In our case, direct and prolonged contact between primary human osteoblasts and material containing Sr-ACP granules confirmed this partial inhibitory effect. In addition, the structure and the great dimension of ACP granules observed by TEM compared to osteoblast cell size, regardless of the presence of Sr ion, could have hampered regular cell division, spreading, and motility, resulting in a lower cell number. Additionally, specific topographical features on the biomaterial surfaces have been shown to be able to drive cell behavior [40].

Proliferation increases in a manner that is inversely proportional to the stage of differentiation [41,42]. Here, we observed that the expression of markers associated with early osteoblast differentiation, *RUNX2*, *SP7*, and *ALPL*, were less expressed on OC+Sr-ACP compared to OC+BMP-2 and OC. This observation could lead to the hypothesis that the cells on OC+Sr-ACP were in a more advanced differentiation stage and, therefore, less proliferating.

NHOsts on OC+BMP-2 showed a higher expression of *RUNX2* when compared to other scaffolds at both timepoints, also showing a very strong expression of *SP7*. BMP-2 confirmed its crucial role in stimulating *RUNX2* expression, in accordance with Halloran and Yun [43,44]. *RUNX2* is a “master” regulator of osteogenesis, belonging to the Runx family of transcription factors that induce the expression of *SP7*, the other osteoblast-specific transcription factor, belonging to the Krüppel-like family; *RUNX2*-mediated activation of *SP7* occurs by binding to an upstream enhancer [45,46].

*SP7*, also known as *Osterix* (*OSX*), supports the maturation of functional osteoblasts, following closely behind the action of *RUNX2.* Interestingly, BMP-2 stimulus can even bypass the osteoblast differentiation triggered by *RUNX2* and maintain the expression level of *OSX* mediated by DLX5 [39]. Our results could suggest that the NHOst on OC+BMP-2 were in a state of not-yet-complete maturation, and their activity was mainly driven by *SP7*.

When developing new scaffolds for bone regeneration, the effects of the material on osteoclasts generally receive less attention. Here, we demonstrated a higher number of osteoclasts forming in the presence of OC+SR-ACP after 14 days via TRAP staining. This is in line with the higher expression of *ACP5* at day 7 and *CTSK* at day 14 compared to the OC control. In the finely orchestrated balance between osteoblasts and osteoclasts activity, aimed at preserving bone health in vivo, the RANKL/RANK/OPG pathway is one of the main systems dedicated to remodeling. The RANKL/RANK pathway triggers osteoclast activity and formation, while OPG (also called RANKL’s decoy receptor) plays a role as a bone-protective factor by binding to RANKL, thus inhibiting osteoclast formation and resorption [47,48]. The process of osteoclastogenesis is known to be attributable to a signaling pathway that begins with RANK/RANKL binding, continues with intracellular interaction between RANK and TRAF6 factor, and ends with subsequent activation of the downstream NF-kB complex. Nevertheless, compelling evidence supports the idea that other co-stimulatory signals could be necessary for osteoclast differentiation and activation: the *RANK/RANKL* complex is part of a positive feedback loop involving the transcription factor *OSCAR*, which is expressed after the establishment of the link between its promotor and activated factors downstream of *RANK/RANKL*, as described in a review by Nedeva [49]. Our observations have therefore labeled *OPG*, *RANKL,* and *OSCAR* as main players of osteoclastogenesis in this co-culture system. The presence of osteoblasts in the microenvironment could be decisive for their release of RANKL and/or OPG, thus differently influencing the activity of osteoclasts, in synergy with the materials. Therefore, the use of mature osteoblasts allowed us to precisely outline that osteoblast/osteoclast crosstalk that is crucial in bone remodeling.

In this co-culture set-up, the presence of Sr ions, incorporated in ACP granules at the concentration of 2.49 wt% [17], probably did not exert inhibitory effects on osteoclastogenesis, as other authors have observed [50,51]. It is likely that NHOst, which maintained a good level of differentiation and activity in the presence of OC+Sr-ACP, activated a balancing mechanism between osteoinduction and osteoclastogenesis, also explained through the *OPG*/*RANKL* ratio. This indicator indeed showed higher values on OC+Sr-ACP than on BMP-2-enriched scaffolds, on which the other osteogenetic markers (such as *RUNX2*, *SP7*, *ALPL*, and *COL1A1*) were expressed at higher levels. Furthermore, the presence of ACP granules may also have been a stimulus for the osteoclasts. In any case, enhanced osteoclast activity can be regarded as beneficial, because scaffold resorption within an adequate period of time is a fundamental part of the regeneration process, which should be driven to the “restitutio ad integrum” of the involved tissue [52].

Finally, *VEGFA* did not increase over time in any materials, but it was less expressed on the enhanced ones, thus providing evidence against a pro-angiogenic role for the scaffold modifications on the osteoblasts. This data should be considered preliminary to a future in-depth analysis of the angiogenesis, because this aspect is essential both for normal bone physiology and in the processes of bone regeneration in which these types of biomaterials are involved [53]. It will be interesting to demonstrate whether, in this context, the angiogenesis, obviously investigated through the analysis of several mediators, will be more attributable to other cell types (e.g., MSCs) or whether it will be expressed at longer NHOst culture times.

A tri-culture system represents an advanced in vitro model for testing bone cells’ biological response to modified scaffolds and their crosstalk. Nevertheless, a dynamic model providing mechanical stimulation capabilities would be even more appropriate. Furthermore, a functional test on osteoclasts, i.e., their resorptive activity, could complete the evaluation.

## 5. Conclusions

Adding both BMP-2 and Sr-ACP to the subchondral bone-like layer of the Col/Col-Mg-HA osteochondral scaffold appears to improve its osteoinductive potential, which was the principal aim of the modifications carried out on the scaffolds, thus confirming what was observed in vivo from the studies of Xu. By using a culture model composed of different cell types, we demonstrated different effects of the materials on primary human osteoblasts and osteoclasts. OC+BMP-2 provided a more targeted stimulus to NHOst, while OC+Sr-ACP induced a stronger overall activation of osteoclasts, thereby enhancing remodeling. Both mechanisms contribute positively to the osteochondral regeneration of this biomaterial.

Future research should be focused on targeting clinical translation of these biomaterials to achieve complete subchondral bone recovery. The current challenge could be to implant the modified materials in order to achieve better results in OA treatment.

Furthermore, we envision that a material enriched with both BMP-2 and Sr-ACP could combine the positive properties of the materials in one single approach.

## Figures and Tables

**Table 1:** Details on the qPCR assay. Sequence of primers, amplicon length, and annealing temperatures used for the evaluation of gene expression by qPCR.

GENE	Primer Forward	Primer Reverse	Amplicon Length	Annealing Temperature
*ACP5*	5′-GAAGCGCAGATAGCCGTT-3′	5′-GGTCACTGCCTACCTGTG-3′	148 bp	60 °C
*ALPL*	QuantiTect Primer Assay (Qiagen) Hs_ALPL_1_SG		110 bp	55 °C
*BGLAP*	QuantiTect Primer Assay (Qiagen) Hs_BGLAP_1_SG		90 bp	55 °C
*COL1A1*	QuantiTect Primer Assay (Qiagen) Hs_COL1A1_1_SG		118 bp	55 °C
*CTSK*	QuantiTect Primer Assay (Qiagen) Hs_CTSK-1_SG		105 bp	55 °C
*GAPDH*	QuantiTect Primer Assay (Qiagen) Hs_GAPDH_1_SG		95 bp	55 °C
*OPG*	QuantiTect Primer Assay (Qiagen) Hs_TNFRSF11B_1_SG		107 bp	55 °C
*OSCAR*	QuantiTect Primer Assay (Qiagen) Hs_OSCAR_1_SG		137 bp	55 °C
*RANKL*	QuantiTect Primer Assay (Qiagen) Hs_TNFSF11_1_SG		91 bp	55 °C
*RUNX2*	QuantiTect Primer Assay (Qiagen) Hs_RUNX2_1_SG		101 bp	55 °C
*SP7*	QuantiTect Primer Assay (Qiagen) Hs_SP7_1_SG		120 bp	55 °C
*SPARC*	QuantiTect Primer Assay (Qiagen) Hs_SPARC_1_SG		60 bp	55 °C
*SPP1*	QuantiTect Primer Assay (Qiagen) Hs_SPP1_1_SG		115 bp	55 °C
*VEGFA*	QuantiTect Primer Assay (Qiagen) Hs_VEGFA_6_SG		99 bp	55 °C

## Data Availability

The original contributions presented in the study are included in the article, further inquiries can be directed to the corresponding author.
